# Research on the Coupled Bionic Design and Validation of Flying Car Folding Wings Based on Eurasian Eagle‐Owl Wing Shape

**DOI:** 10.1155/abb/7445905

**Published:** 2025-12-20

**Authors:** Zhengjun Li, Yuchen Cao, Dehao Zhao

**Affiliations:** ^1^ School of Design and Art, Shenyang Aerospace University, Shenyang, 110000, China, sau.edu.cn

**Keywords:** aerodynamic performance, coupled bionic design, Eurasian eagle-owl wing shape, flying car wings, numerical simulation

## Abstract

**Objective:**

This study explores the application of Eurasian eagle‐owl wing characteristics to the design of folding wings for flying cars. By analyzing the aerodynamics of the eagle‐owl wing, we aim to innovate folding wing configurations to improve lift, reduce drag, enhance flight stability, and ultimately increase the overall energy efficiency and safety of flying cars.

**Methods:**

First, a comparative analysis of aerodynamic performance data across multiple owl species was conducted, leading to the selection of the Eurasian eagle‐owl wing as the bionic prototype. Then, reverse engineering modeling was performed using image‐based photogrammetry. A three‐dimensional shape error measurement method was applied for quantitative error analysis of the reconstructed model. High‐precision point cloud data of the wing were obtained and sliced at equal intervals. The extracted airfoil cross‐sections were fitted using polynomial equations and simulated in XFOIL. Sections exhibiting superior aerodynamic performance were selected as bionic airfoils. Next, using coupled extension analysis method and a comprehensive coupling degree evaluation function from coupled bionics, the coupling bionic feature vectors and eigenvalues between the folding wing and the bionic reference were analyzed. A coupled extension matrix model was established to guide the bionic design based on eagle‐owl wing morphology. Finally, fluid simulations were performed using Fluent software, and a comparative analysis of aerodynamic performance was conducted.

**Results:**

The results reveal that the folding wing design inspired by the Eurasian eagle‐owl significantly improves lift, reduces drag, and enhances flight stability compared to traditional wing designs.

**Conclusion:**

The bionic design of flying car folding wings based on the Eurasian eagle‐owl wing proves effective in enhancing aerodynamic performance.

## 1. Introduction

Flying cars, capable of both aerial flight and terrestrial travel, are now considered one of the most versatile and efficient future transportation solutions. As such, their development is advancing globally. For example, Terrafugia’s Transition completed a successful test flight in 2009; AeroMobil of Slovakia launched the first mass‐produced flying car in 2017; and in 2021, China’s XPeng Huitian released the two‐seater intelligent electric aircraft Traveler X2 alongside a sixth‐generation flying car concept [1]. This paper builds upon the current folding wing design of the Ruixiang flying car and conducts optimization research through coupled biomimetic methods. It presents a study on the coupled biomimetic design and validation of flying car folding wings based on the Eurasian eagle‐owl wing shape, aiming to improve aerodynamic efficiency, stability, safety, and adaptability across various application scenarios.

### 1.1. Research Content and Process

First, a theoretical foundation and literature review were established. This involved analyzing theories of coupled bionics and computational fluid dynamics (CFDs) to establish the theoretical and methodological foundation for this study. Existing studies on coupled bionic design, particularly for flying car folding wings, were probed to summarize findings, identify research gaps, and clarify the significance of this work. Next, the research methods were structured in three stages: The first step involves data collection for the coupled bionic design of flying car wings, gathering data on the Eurasian eagle‐owl wing shape as the biomimetic subject and data on the Ruixiang flying car and its wings to lay the groundwork for subsequent practical application. The second step is the coupled analysis method for the biomimetic design of flying car wings, which includes bio‐coupled element extension analysis, bio‐coupling mode extension analysis, and constructing the bio‐coupled extension matrix model to define the characteristic vectors and eigenvalues of the coupling elements. The third step is the coupled bionic design and fluid simulation validation of the flying car folding wings, involving mesh preprocessing, boundary condition setting, turbulence model selection, numerical simulation, and post‐processing analysis. Finally, practical research was conducted: first, the folding wing was designed using eagle‐owl wing data; second, CFD simulations were performed to verify and optimize aerodynamic performance, ensuring design reliability.

### 1.2. Research Conclusions and Significance

Empirical research demonstrates that the coupled bionic design of flying car folding wings based on the Eurasian eagle‐owl wing shape effectively improves performance in several ways: increased lift, improved gliding safety, enhanced landing stability, and reduced energy consumption, leading to higher flight efficiency. Thus increasing flight efficiency. This study explores a practical path for the integrated optimization of flying car wings in terms of aerodynamics, stability, safety, and adaptability, and offers guidance for future innovative designs.

## 2. Theoretical Foundations and Literature Review

### 2.1. Theoretical Foundations

#### 2.1.1. Coupled Bionics

Coupled bionics is one of the latest developments in bionics and a significant part of it. It facilitates the transition from single‐element to multi‐element imitation, from morphological similarity to functional equivalence, and from single‐domain studies to integrated multi‐physics approaches spanning geometry, physics, chemistry, and biology. Research on synergistic mechanisms and principles of factors such as morphology and structural materials in biological coupling, and the establishment of a universally applicable biological coupling model, are key to construct a comprehensive theory of bionic coupling that integrates form, structure, material, and function [2]. The main content of the study includes two major parts: biological coupling analysis and biomimetic coupling design. The former includes analyzing biological performance, behavior, and coupling elements and modes; studying functional principles and formation mechanisms; and performing modular and extension analyses. The latter involves principles, methods, and processes of bionic coupling design; development of bionic functional products; and performance evaluation. This article relies on the theoretical and methodological foundation of coupled bionics, combining coupling elements and coupling models to carry out extension analysis and complete the biomimetic coupling design.

The coupling element and the coupling mode are the core concepts that constitute the theoretical system of coupled design. The coupling element consists of matter‐elements and affair‐elements, while the coupling mode is expressed through relation‐elements. An affair‐element describes an event using a triple (event name, feature vector, feature value). In this study, the affair‐element is used to represent the coupled bionic design scheme of the folding wing of a flying car, where *O*_*a*_ denotes the event name referring to the research object, *C*_*a*_ is the feature vector describing the attribute characteristics of the event, and *V*_*a*_ signifies the feature value quantifying the corresponding attributes. This ternary structure clearly defines the fundamental components of a system, providing a theoretical foundation for the structured representation of complex systems. A matter‐element is the basic unit used to describe the name, characteristics, and feature values of an objective entity. In this study, the constructed matter‐elements include the morphological features of the “new concept” aircraft and the Eurasian eagle‐owl wing. Here, *O*_*m*_ denotes the name of the object, identifying the specific target entity; *C*_*m*_ represents a particular characteristic of the object, used to describe its structural, functional, or performance‐related attributes; and *V*_*m*_ refers to the specific value or parameter of the characteristic under certain conditions, serving to quantify the performance of the characteristic within the research context. describes relationships between entities as a triple (relation name, relation characteristic, value). In this research, the relation‐element is modeled using fluid resistance formulas. *O*_*r*_ denotes the relation name, indicating the specific connection between the affair‐element and the matter‐element; *C*_*r*_ represents the relation characteristic, which describes the associated attributes between the two (such as morphological similarity or coupling mode); and *V*_*r*_ is the specific value of the relation characteristic, used to quantify the form or state of the relation under specific conditions.

#### 2.1.2. CFDs

CFD emerged in the 1960s as an interdisciplinary field combining computer science, fluid mechanics, mathematical theory of partial differential equations, computational geometry, and numerical analysis. CFD numerically solves fluid flow control equations by discretizing them over a computational grid. It is based on the fundamental laws governing fluid flow (laws of conservation of mass, momentum, and energy), constructing continuity equations, momentum equations, and energy equations, and solving them using numerical methods. The solution process begins by specifying the geometric shape and dimensions of the model, defining the computational domain, and setting appropriate boundary conditions for inlets, outlets, walls, and free surfaces. It also requires suitable mathematical models and a complete mathematical description of the process equations, including initial conditions. The main solution methods include: Finite Difference Method (FDM), Finite Element Method (FEM), and Finite Volume Method (FVM) [3]. Fluent software was adopted in this work as a numerical simulation tool to study the lift and drag reduction effects on the wings of flying cars.

### 2.2. Literature Review

#### 2.2.1. Overview of Research on Coupled Bionic Design

Literature review shows that coupled bionic design research focuses on five areas: material‐coupled bionic design, structural coupled bionic design, power system coupled bionic design, functional coupled bionic design, and morphological coupled bionic design. Material‐coupled bionic design: Zhang et al. [4] designed bionic materials with erosion and wear resistance inspired by razor clam shells, improved via laser processing. Power system‐coupled bionic design: Zuo et al. [5] studied a dynamics model of a rigid‐flexible coupled bionic robotic fish, validating a feasible approach for body curve analysis. Structural‐coupled bionic design: Xie et al. [6] combined wavy leading edges, serrated trailing edges, and surface ridges on a NACA0018 airfoil to form a WSR‐type airfoil and studied its noise reduction mechanism. Functional‐coupled bionic design: Mei et al. [7] studied the motion characteristics of cat claws in different gaits to guide the bionic design of tire treads, thereby optimizing tire noise and grip performance. Morphological‐coupled bionic design and methods: Xu et al. [8] coupled biological prototypes with product design using esthetic, functional, and semantic methods, applying fuzzy comprehensive evaluation to select optimal schemes. Chen et al. [9] utilized orthogonal optimization design to optimize the wavelength and amplitude of the non‐smooth leading edge of an owl wing profile, extracting the cross‐sectional characteristics and nonsmooth leading edge shape of the airfoil, based on the orthogonal optimization results to determine the optimal combination of wavelength, amplitude, and airfoil type for the design of blades for horizontal‐axis wind turbines. Xu et al. [10] applied reverse reconstruction technology to quantify the geometric information of the leading edge morphological characteristics of the long‐eared owl’s wing feathers and established a biomimetic coupling model. They used the SIMPLEC algorithm based on the FVM and pressure correction to numerically simulate the aerodynamic characteristics of the biomimetic‐coupled airfoil model [10].

The aforementioned studies indicate the successful application of coupled bionics across domains, affirming its effectiveness. Their methodologies can be extended to the study of folding wings for flying cars. Previous morphological studies provide clear technical pathways, especially in airfoil data acquisition and fluid simulation, for this work. As a nascent area, bionic folding wing research for flying cars remains underexplored, highlighting the practical significance of this study.

#### 2.2.2. Overview of Research on Coupled Bionic Design of Folding Wings for Flying Cars

The overview of research related to the coupled bionic design of folding wings for flying cars is conducted from two perspectives. Firstly, a literature study on the design of folding wings for flying cars is undertaken, and secondly, research on the coupled bionic design of these folding wings is explored to systematically clarify the progress in the field of flying cars with folding wings. Currently, research on the design of flying car folding wings mainly focuses on two aspects. Mechanical design: exploring the structural design of flying car folding wings. Lin et al. [11] designed folding mechanisms, modeled kinematics using the complex vector method, and simulated motion in Adams software. Yu et al. [12] applied integrated design technology for folding mechanisms and force transmission structures, designing a wing folding mechanism based on the cam‐wheel principle; the analysis and validation of aerodynamic characteristics for folding wing designs. Shao [13] discussed a new tilt‐rotor flying car design and conducted an aerodynamic characteristics analysis. This flying car merges features of rotary‐wing and fixed‐wing foldable aircraft. Through multi‐modal aerodynamic simulation analysis, the design ensures that the flying car meets the set technical specifications [13]. Regarding bionic design, few studies exist, with only one notable publication by Chen et al. [14] titled *Aerodynamic Characteristics Simulation Analysis of a Flying Car Based on Bionic Seagull Wings*, which involves the design of a seagull‐wing car and optimized parameters using a Pareto genetic algorithm.

The research discussed above indicates that the design of folding wings for flying cars primarily originates from the perspective of mechanical principles, exploring the rationality of mechanical structures and the integrated design practices of structural and transmission mechanisms. Aerodynamic analysis of folding wings generally employs fluid simulation for validation. Research findings in the coupled bionic design of flying car folding wings are limited. Existing studies often lack comprehensive research design and rigorous technical pathways. A notable deficiency lies in the application of reverse engineering for data acquisition, and the overall reliability of these investigations requires further validation. Consequently, there is a clear need to explore optimized data acquisition from biomimetic subjects using reverse engineering methods to carry out coupled bionic design research and to clarify the reliability of fluid simulation in the experimental validation of coupled bionic design for flying car folding wings.

Based on the analysis and review of the literature, this study proposes a coupled‐bionic design and validation research of folding wings for flying cars based on the Eurasian eagle‐owl wing shape. Addressing identified research gaps, it introduces innovative methodologies into coupled bionic design. Specifically, a three‐dimensional point cloud model of the owl wing is generated via image‐based photogrammetry. Equidistant slicing analysis and aerodynamic analysis using Xfoil are conducted to select wing sections with superior aerodynamic performance. The morphological curves of these sections are characterized using polynomial fitting equations. Extension analysis determines the characteristic vectors, which are combined with an analysis of coupling modes derived from lift and drag equations. Further optimization of the curves through adjustment of control points enables the morphing of the biomimetic prototype to fit the flying car’s folding wings. The final coupled bionic design undergoes validation through fluid simulation experiments, aiming to comprehensively optimize the flying car’s aerodynamic performance, stability, safety, and adaptability to different application scenarios.

## 3. Research Methods

### 3.1. Data Collection for Coupled Bionic Design of Folding Wings for Flying Cars

#### 3.1.1. Data Collection on the Eurasian Eagle‐Owl Wing Shape as a Biomimetic Model

Based on the scientific data from the Cornell Lab Bird Academy (https://academy.allaboutbirds.org) bird database and in combination with the specific requirements of flying cars, a comparative analysis of the flight data of various owl species was conducted. This analysis led to the selection of the Eurasian eagle‐owl wing shape as the biomimetic model for the folding wings [15].

#### 3.1.2. Reverse Engineering Methods

Three‐dimensional morphological reconstruction of the eagle‐owl wing profile was achieved through reverse engineering, converting two‐dimensional images into point cloud data. Photogrammetry served as the primary data acquisition technique. First, multi‐angle images (top, bottom, front, rear, and right views) of the Eurasian eagle‐owl wing were sourced from the iNaturalist website (Observation · iNaturalist), ensuring coverage of the upper surface, lower surface, leading edge, and trailing edge of the wing. These multi‐angle images were imported into Meshroom software, which was used to generate the three‐dimensional point cloud data from the two‐dimensional images of the wing.

Subsequently, the obtained point cloud model was imported into CATIA software for processing, including merging, simplification, noise reduction, and surface fitting. A smooth surface wing model was then constructed in CATIA by converting the point cloud data into a continuous surface. During this process, surface accuracy was verified and refined using CATIA’s error analysis tools, maintaining a deviation within ±0.05 mm from the original point cloud data.

To evaluate the accuracy of the morphological data in the 3D model of the Eurasian eagle‐owl wing generated via photogrammetry, a three‐dimensional shape error measurement method was employed. The coordinate system was defined with the body length direction as the *X*‐axis, wing width as the *Y*‐axis, and wing height as the *Z*‐axis. The reconstructed model was scaled proportionally based on the actual wingspan of the owl. Standard deviations in the wing width and wing length directions were calculated using the 3D shape error measurement method, and the resulting error data were analyzed to assess the modeling accuracy of the photogrammetry‐based 3D reconstruction of the Eurasian eagle‐owl wing profile.

#### 3.1.3. Equidistant Slicing Analysis

First, airfoil sections are extracted from the reconstructed 3D model of the unfolded wing along the spanwise direction, starting from the wing root. Sections are taken at 10% intervals, producing a total of nine profiles. This interval strikes a balance between data richness and processing complexity; a smaller interval increases computational burden, while a larger one risks losing critical morphological information. For the coupled bionic design, the upper surface curve, leading edge curve, and trailing edge curve of the eagle‐owl airfoil were extracted for the coupled bionic design. The lower surface curve was smoothed to increase the pressure differential between the upper and lower surfaces, thereby enhancing lift generation.

The upper surface contour line of each airfoil section was obtained using the point tracing method, and the coordinates of the upper surface points were acquired. These points were then used to extract the upper surface curves of the airfoil, which were fitted using polynomial equations (see Equation 1). A fourth‐order polynomial was applied to obtain the coordinate values (*x*, *y*) of the upper surface curve. The selection of the polynomial regression model rested on a comprehensive analysis incorporating the comprehensive coupling degree index function and the extension matrix correlation function. This selection also considered the criteria for similarity‐based coupling and the goal of minimizing drag coefficient (CD) in the experimental results.

Specifically, the order of the polynomial fitting was determined according to the complexity of the eagle‐owl airfoil profile curves. Regions exhibiting smooth morphological variations were fitted with lower‐order polynomials to ensure stability, whereas complex regions require higher‐order polynomials for accuracy, while lessening potential overfitting and oscillatory behavior. Finally, the aerodynamic performance of the nine airfoil sections was analyzed using XFOIL software, and section screening was conducted to provide a basis for the coupled analysis of the bionic reference.

(1) y=anxn+⋯+a2x2+a1x+a0.

In the formula, *x* represents the chord coordinates of points on the upper wing surface, *a* is the variable coefficient for the coordinates, and *y* signifies the curve of the upper wing surface.

#### 3.1.4. Data Collection Related to Folding Wings of Flying Cars

Relevant data of the Ruixiang flying car and its folding wings were gathered; the specifics are presented in Table 1.

**Table 1:** Ruixiang flying car folding wing‐related data.

Name	Wingspan	Wing chord length	Length of the flying car	Width of the flying car (after wing folding)
Ruixiang flying car	8 m	0.9 m	6 m	2.3 m

### 3.2. Coupled Analysis Methods for Biomimetic Design of Flying Car Wings

#### 3.2.1. Bio‐Coupled Element Extension Analysis

Affair‐element analysis. Based on the relevant data of the flying car folding wing presented in Section 3.1.4, an extension analysis of the affair‐element for the coupled bionic design scheme of the folding wing was conducted, yielding the establishment of extension matrix model *A* = (*O*_*a*_, *C*_*a*_, *V*_*a*_). In this model, *A* represents the affair‐element, *O*_*a*_ denotes the object of the extension analysis, *C*_*a*_ is the feature vector, and *V*_*a*_ represents the feature value corresponding to the feature vector.

To ensure the rationality of the feature vectors and feature values within the affair‐element’s extension matrix, a Weight Vector *w* = [*w*_1_, *w*_2_……*w*_*n*_] was introduced to signify the relative importance of different features in the coupling decision. The G1 method was used to determine these weights. Ten experts with relevant professional backgrounds were invited to comprehensively evaluate the coupling feature data of matter‐element *M*_1_, *M*_2_ and assign appropriate weights to each indicator, as shown in Equation (2). This provides a quantitative basis for subsequent calculation of the comprehensive coupling degree index between affair‐elements and matter‐elements.
(2)

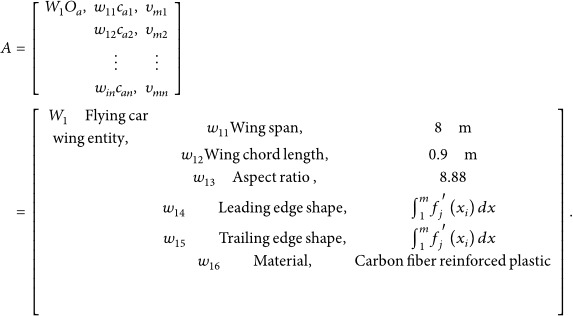




Based on the data of the new concept flying car folding wing, a coupling element (coupling‐unit) analysis of the folding wing was conducted. This involved first analyzing the feature vectors within the folding wing’s coupling element, followed by acquiring the corresponding feature value parameters. These values served as optimization targets and dimensional constraints. The extension matrix model of the coupling element for the flying car wing is presented in Equation (3) [2].
(3)

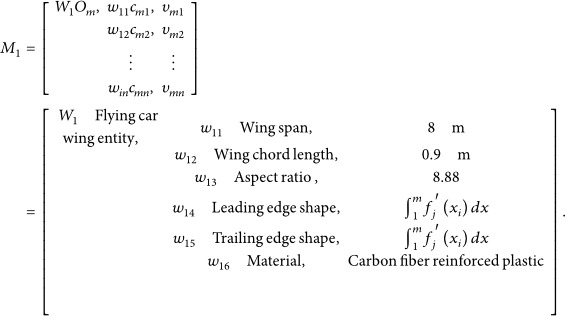




Based on the screened airfoil sections from the Eurasian eagle‐owl wing model, an extension analysis of the biological coupling element was conducted. First, the feature vectors of the biological coupling element were analyzed, which included the cross‐sectional geometry, airfoil shape, and airfoil dimensions. Then, the corresponding feature values of these feature vectors were examined. *W* represents the weights assigned to the indicators of matter‐element *M*_1_, *M*_2_ by 10 experts with relevant professional backgrounds using the G1 method. The extension matrix model of the coupling element for the eagle‐owl wing is shown in Equation (4) [2]

(4) M2=W1Om1w11cm1vm1 w12cm2vm2 ⋮⋮ wincmnvmn=W1 Eurasian owl wing morphology elements, w11 Trimmed wing length,vm1w12 Trimmed wing chord length,vm2w13 Aspect ratio,vm3w14 Airfoil cross-sectional profile,∫1mfj′xi dxw15 Leading edge shape,∫1mfj′xi dxw16 Trailing edge shape,∫1mfj′xi dx.

Comprehensive Coupling Degree Index Evaluation Between Affair‐Element and Matter‐Element. A coupling degree evaluation function (Equation (5)) was constructed in this work to quantitatively assess the coupling between the design parameters (affair‐element) of the flying car folding wing and the morphological parameters (matter‐element) of the eagle‐owl wing. This function defines similarity evaluation criteria, transforming the qualitative extension coupling matrix into a quantitative mapping. Comprehensive coupling degree evaluation function:

(5) C=∑i=1nwi⋅Kixi.

In the equation, *C* denotes the coupling degree. The standard criterion for similarity‐based coupling *C* *＞*0.5 (with specific values determined experimentally based on the minimization of the CD) indicates a high degree of coupling between the coupled bionic morphology of the flying car folding wing and the morphological features of the Eurasian eagle‐owl wing. *w*_*i*_ represents the weight of the *i*th feature vector. The term *K*_*i*_(*x*_*i*_) refers to the correlation function of the affair‐element feature vector, as defined in Equation (6).

(6) Kixi=x−ab−a, a≤x≤bx−ba−b,x<aorx>b.

In the equation, *x*_*i*_ represents the feature value of the component in the feature vector; [*a*, *b*] denotes the interval of the biological feature value *v*_*m*_. Correlation increases as *K*_*i*_(*x*_*i*_) approaches 0 or 1. However, values approaching the negative extreme indicate significant deviation from the coupling relationship. Under such conditions, polynomial regression may be prone to overfitting and oscillatory behavior.

#### 3.2.2. Extension Analysis of Biological Coupling Methods

Coupling Methods Analysis Using Relation‐Elements: Coupling methods, which describe how biological coupling elements interact to achieve specific functions, are formalized using relation‐elements in extension theory. In this study, the relation elements are aerodynamic equations, namely Bernoulli’s equation, lift equation, and drag equation [16]. These equations are represented in the coupled extension matrix model as relations Equation (7) through Equation (9):

(7) R1=or,cr1,vr1 cr2,vr2 ⋮⋮ crnvrn=p+12ρv2+ρgh=constantp pressurevr1 ρ densityvr2 g  gravitational accelerationvr3 h heightvr4,

(8) R2=Or,cr1,vr1 cr2,vr2 ⋮⋮ crn,vrn=L=CL12ρv2AL liftvr1 CL lift coefficientvr2 ρ air densityvr3 v velocity relative to the objectvr4 A reference areavr5   ,

(9) R3=Or,cr1,vr1 cr2,vr2 ⋮⋮ crn,vrn=D=CD12ρv2AD dragvr1 CD drag coefficientvr2 ρ air densityvr3 v relative flow velocityvr4 A characteristic area of the objectvr5   .

#### 3.2.3. Establishment of the Bio‐Coupled Extension Matrix Model

Integrating the coupled element extension model and the coupling method extension model outlined above, the following biological coupling model is established as Equation (10):

(10) B=Biomimetic coupling, Function cm1, Vm1 Coupling element cm2,M1∧M2…Mi Coupling method cm3,R01⊕R02⊕⋯⊕Rn Work environment cm4,Vm4=Biomimetic coupling, Function,Lift performance∧Drag reduction performance Coupling element,Eurasian owl wing shape coupling element∧Flying car folding wing coupling element Coupling method,Lift equation⊕Resistance equation⊕Bernoulli′s equation Work environment, Low altitude environment.

The aforementioned model is the bio‐coupled extension matrix model *B* in coupled bionics. For the specific case of designing folding wings for flying cars based on the Eurasian eagle‐owl wing, the function *c*_*m*1_ includes lift performance and drag reduction performance; the coupled elements *c*_*m*2_ are divided into the folding wing coupled elements of the flying car and the morphological coupled elements of the Eurasian eagle‐owl wing shape; the coupling method *c*_*m*3_ consists of aerodynamic equations, specifically the lift equation, drag equation, and Bernoulli’s equation; and the operating environment *c*_*m*4_ is the low‐altitude environment.

### 3.3. Coupled Bionic Design of Folding Wings for Flying Cars

The aforementioned bio‐coupled extension matrix model serves as the foundation for the coupled bionic design. A geometric model was created using CAD software and subsequently simplified in Rhino. This involves removing small‐sized features and internal structures that do not bear load, while preserving external features critical to aerodynamic performance, thus reducing computational complexity. The final model is saved in STP format for import into Fluent.

### 3.4. Fluid Simulation Validation of Folding Wing Design for Flying Cars

#### 3.4.1. Determining the Computational Domain

The computational domain size was set to fully encompass all critical flow regions. Boundary conditions were established for the inlet, outlet, walls, and symmetry planes.

#### 3.4.2. Mesh Generation

An appropriate mesh type was selected based on flow complexity and geometric characteristics. An unstructured mesh was adopted in this study. Global mesh generation was then performed, and the mesh size was adjusted to meet overall computational requirements. Local refinement was applied in regions with sharp gradients in flow properties, such as boundary layers, wake regions, and fluid–solid interfaces. Following these steps, a final check and a mesh independence study were conducted to ensure the mesh density adequately resolved complex flows. Meshing was performed within Flent, and the final mesh was exported in a CFD‐compatible format for numerical solving.

#### 3.4.3. Setting Boundary Conditions and Choosing Turbulence Models

For the fluid simulation of the folding wing, appropriate boundary conditions and a suitable turbulence model must be defined. Boundary conditions are specified precisely according to the physical scenario: the inlet boundary typically requires the specification of fluid velocity, temperature, and turbulence parameters; the outlet is set as a pressure outlet or free outflow to simulate actual flow conditions, while solid wall boundaries are set as no‐slip wall conditions. The choice of turbulence model should be based on the characteristics of the flow and the specific objectives of the simulation. In this study, the *S* *S* *T* *k* − *ω* model was selected, which combines the strengths of the *k* − *ε* and *k* − *ω* models, offering superior performance for near‐wall flows and improved prediction of flow separation.

#### 3.4.4. Numerical Calculation Results and Post‐Processing

First, a pressure‐velocity coupled solver was used with the SIMPLE algorithm for the incompressible flow around the wing. This solver coupled pressure and velocity fields semi‐implicitly and ensured the stability and accuracy of the computations. Discretization schemes with at least second‐order accuracy in space were applied to enhance the capture of flow details, particularly in separation and reattachment regions. The number of iterations was set to ensure temporal accuracy and convergence. Initial and boundary conditions were precisely set based on experimental data or theoretical predictions to replicate real‐world operating conditions. Residual reduction and key physical quantities were monitored throughout the simulation, with solution parameters adjusted as needed to address potential numerical instabilities [17]. Key flow field data were obtained from Fluent, including lift and drag data related to the folding wings of flying cars at different angles of attack and flow velocities. Subsequently, data visualization was performed, including pressure contours, velocity distributions, and velocity vector plots, to visually display the fluid dynamics on and around the wing surfaces.

#### 3.4.5. Data Analysis

The lift coefficient (LC), CD, and lift‐to‐drag ratio data for the folding wing across different angles of attack and flow velocities were analyzed with reference to the performance ranges suggested by Daniel [18]. According to Jiri Blazek’s [19] criteria for evaluating pressure contour maps, velocity distribution maps, and velocity vector maps, these graphical data were analyzed. This analysis aimed to determine whether the coupled bionic design of the folding wing, inspired by the Eurasian Eagle‐Owl, met the predefined aerodynamic performance objectives.

## 4. Research Practice

### 4.1. Data Collection for Coupled Bionic Design of Flying Car Wings

#### 4.1.1. Selection of Biomimetic Subject

A detailed comparative analysis of flight and wing shape data among owl species was conducted using scientific data from the Cornell Lab Bird Academy and related literature [20–23]. The results, summarized in Table 2, indicate the superior flight performance data associated with the Eurasian eagle‐owl wing shape, making its selection as the biomimetic prototype for the flying car folding wing design.

**Table 2:** Owl species wing shape data.

Name	Wingspan (m)	Wing width (m)	Wing chord ratio	Area (m^2^)	Lift coefficient	Drag coefficient	Flight speed(km/h)	Glide ratio
Long eared owl	0.9–1	0.15–0.2	16	0.135	0.55	0.08	20–40	6.875
Collared owl	0.46–0.61	0.14–0.17	8.6	0.0644	—	—	20–40	—
Barn owl	0.8–0.95	0.145–0.175	12.9	0.116	0.7	—	40–50	—
Eagle owl	1.6–1.88	0.378–0.518	11.7	0.6048	0.896	0.1	50–60	8.96
Great horned Owl	1.01–1.53	0.305–0.375	11.7	0.308	0.33	0.07	60	4.7

#### 4.1.2. Three‐Dimensional Reconstruction of the Eurasian Eagle‐Owl Wing Shape Using Reverse Engineering Techniques

**Figure 1:**
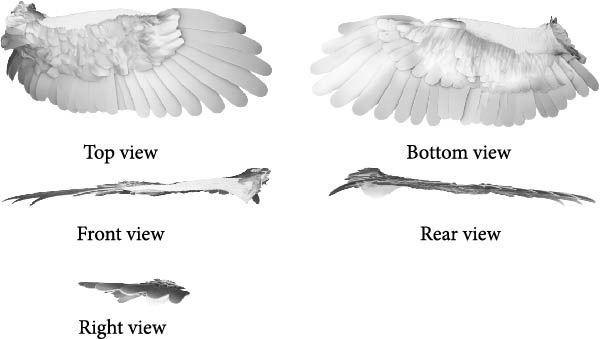
Five views of the Eurasian eagle‐owl wing shape.

The 3D morphology of the expanded gliding wing of the Eurasian eagle‐owl was reconstructed using image‐based photogrammetry. Multi‐view images (top, bottom, front, rear, and right) covering the wing’s upper and lower surfaces and leading and trailing edges (Figure 1) from iNaturalist were processed in Meshroom software. Adjustments were made to the camera initialization, feature extraction, image matching, feature matching, structure from motion, dense scene reconstruction, depth mapping, depth map filtering, mesh generation, and texture mapping node parameters to reconstruct the three‐dimensional model of the Eurasian eagle‐owl wing shape, which was then saved in STL format.

**Figure 2:**
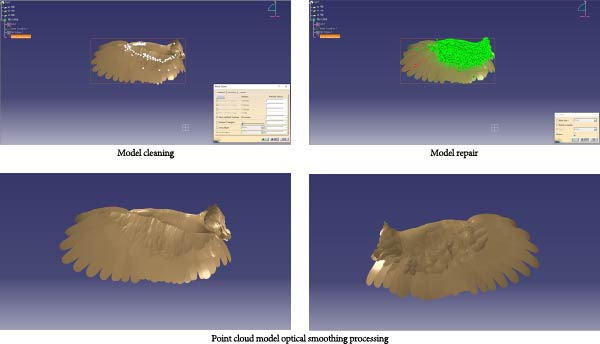
Model cleaning, model repair, and point cloud model smoothing.

The three‐dimensional point cloud model was imported into CATIA software for merging, simplification, denoising, and fitting processes. (1) Input point cloud: The point cloud was imported via the DSE workbench. (2) Merging point clouds: As the original model was a single entity, merging was primarily achieved using the “Merge Clouds” command. (3) Point cloud filtering and noise removal: The “Filter” command (adaptive mode chosen for feature preservation) and the “Remove” command were used for filtering and noise reduction. (4) Meshing the point cloud: The “Mesh Creation” command was applied to mesh the point cloud model, generating a point cloud mesh. The “Mesh Cleaner” was then adopted to clear any meshing issues present on the point cloud model. The “Fill Holes” function filled gaps in the mesh surface to maintain surface integrity. The “Flip Edges” command for refining the model was then used to make it more accurate. (5) Smoothing the point cloud: Finally, the “Mesh Smoothing” command was applied with results shown in Figure 2.

**Figure 3:**
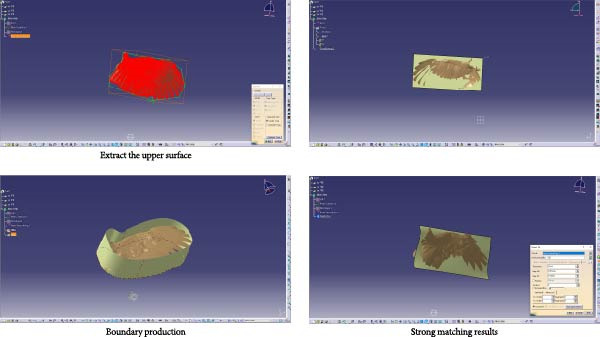
Upper surface extraction, robust matching results, and boundary creation.

**Figure 4:**
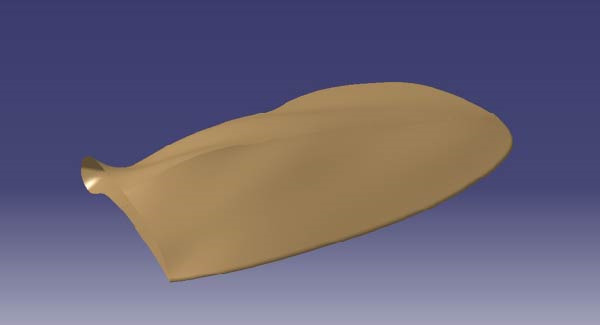
Surface model construction.

Surface construction continued in CATIA. The upper surface point cloud was activated (DSE workbench) and fitted into a surface using the “Power Fit” command (QSR workbench). Local fine‐tuning was performed using the “Control Points” command (FreeStyle workbench) to ensure precision against the point cloud. The surface model was further optimized through flanging and edge operations (Figure 3). Using CATIA’s error analysis feature, deviations between the computed surface and the original point cloud data were analyzed to ensure errors for the surface model were controlled within *a* ±0.05 mm range, with results shown in Figure 4.

The accuracy of the photogrammetrically derived 3D model was assessed quantitatively using a 3D shape error measurement method. The coordinate system was defined with the wingspan direction as the *X*‐axis, the chordwise direction as the *Y*‐axis, and the vertical direction as the *Z*‐axis. The model was scaled proportionally based on the actual eagle‐owl wingspan.

Subsequently, the standard deviations of coordinate points along the X and *Y* axes were measured using the 3D shape error measurement method. The lateral view of the owl’s wing was aligned with the tip of the 3D model at the origin (0, 0, 0), and equidistant slices were taken at 20% intervals. Point coordinate data were extracted using Rhino software.

Finally, the coordinate data from both the physical image and the 3D model were subjected to error analysis to assess the modeling accuracy of the photogrammetry‐based 3D reconstruction of the Eurasian eagle‐owl wing morphology, summarized in Table 3.

**Table 3:** Error statistics table for 3D model generated by photogrammetry.

Interval	Biological point coordinates	3D model point coordinates	Standard deviation	Mean error
20%	(93,313) (93,−36)	(93,314) (93,–37)	0.56%	—
40%	(280,363) (280,–45)	(280,364) (280,–46)	0.48%	—
60%	(467,383) (467,–21)	(467,384) (467,–23)	0.73%	0.55%
80%	(655,378) (655,27)	(655,377) (655,29)	0.24%	—
100%	(748,320) (748,78)	(748,322) (748,79)	0.74%	—

The average error of the equidistant slice point coordinate data along the *X–Y* axes was 0.55%, indicating that the model falls within a reasonable error margin and exhibits high geometric fidelity.

#### 4.1.3. Equidistant Slicing Analysis of the Eurasian Eagle‐Owl Wing Shape Model

**Figure 5:**
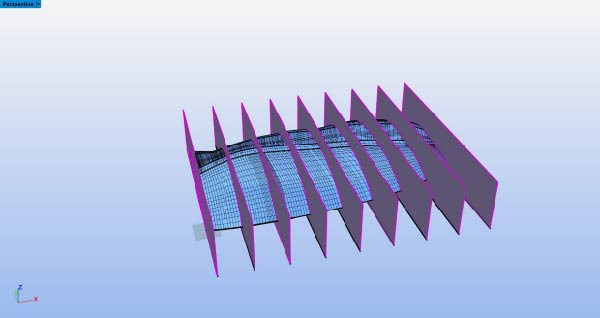
Airfoil cross‐section slicing.

**Figure 6:**
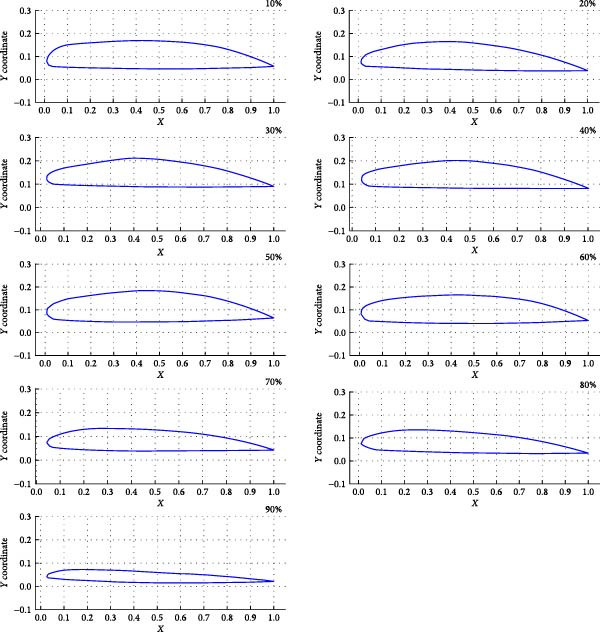
Airfoil curves at the 10%–90% cross‐sections.

The processed 3D point cloud model of the expanded Eurasian Eagle‐Owl wing shape was imported into Rhino software. Using the wing root as the endpoint and the spanwise direction as the axis, the “Contour” command was used to extract cross‐sections at 10% intervals, yielding nine wing cross‐sections, as shown in Figure 5. In Rhino, the “Add Points” command was used to plot points along the upper surface contour line of the wing at the sections, and the coordinate data was exported in TXT format to obtain the upper surface point coordinates. The “Duplicate Edge” command was then applied to extract the curve of the wing’s upper surface, combined with a fourth‐order polynomial fitting equation (fitting relationship equations: Equations (11)–(19)) executed in Python using the polyfit function. The upper surface curve of the wing, smoothed by polynomial fitting, provides an ideal model for aerodynamic performance analysis. The lower surface curve of the wing is smoothed, and the wing curves are plotted using the pyplot module from the matplotlib library in Python software. The final wing‐shape curve, as shown in Figure 6, is obtained.

The fitting relationship for the upper surface curve of the wing section at 10% wingspan is shown in Equation (11):

(11) Y=−5.4657.9444.4020.920486.44×10−9X4+×10−6X3−×10−3X2+X+.

The fitting relationship for the upper surface curve of the wing section at 20% wingspan is presented in Equation (12):

(12) Y=−2.1793.9823.3381.08167.13×10−9X4+×10−6X3−×10−3X2+X+.

The fitting relationship for the upper surface curve of the wing section at 30% wingspan is displayed in Equation (13):

(13) Y=−1.6423.1832.8851.06177.62×10−9X4+×10−6X3−×10−3X2+X+.

The fitting relationship for the upper surface curve of the wing section at 40% wingspan is shown in Equation (14):

(14) Y=−1.5612.8922.4680.903380.10×10−9X4+×10−6X3−×10−3X2+X+.

The fitting relationship for the upper surface curve of the wing section at 50% wingspan is shown in Equation (15):

(15) Y=−1.5922.8872.3800.868761.32×10−9X4+×10−6X3−×10−3X2+X+.

The fitting relationship for the upper surface curve of the wing section at 60% wingspan is displayed in Equation (16):

(16) Y=−1.6912.9662.2830.789852.09×10−9X4+×10−6X3−×10−3X2+X+.

The fitting relationship for the upper surface curve of the wing section at 70% wingspan is shown in Equation (17):

(17) Y=−1.2632.0611.6130.597343.33×10−9X4+×10−6X3−×10−3X2+X+.

The fitting relationship for the upper surface curve of the wing section at 80% wingspan is presented in Equation (18):

(18) Y=−3.2564.8882.8840.726235.70×10−9X4+×10−6X3−×10−3X2+X+.

The fitting relationship for the upper surface curve of the wing section at 90% wingspan is shown in Equation (19):

(19) Y=−1.6381.7446.4770.889237.15×10−9X4+×10−6X3−×10−3X2+X+.

**Figure 7:**
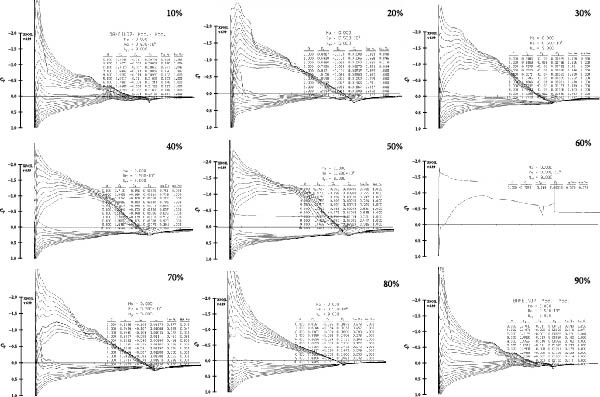
10%–90% airfoil aerodynamic data.

The coordinate data for the nine wing section curves were saved as. dat files. In Xfoil software, the coordinate data files were loaded, and the wing curves were displayed using the “ppar” command. The viscous flow option was activated (“visc” command) with a Reynolds number of 500,000 and 100 iterations. The “as” command was used to set an initial angle of 0°, a final angle of 10°, with an interval of 1°, performing calculations for each of the nine wing sections separately. The aerodynamic data for the wing sections are outputted, as shown in Figure 7. The resulting aerodynamic data revealed that the sections from 10% to 90% span exhibited good performance: the LC increased steadily with the angle of attack, CDs were low, and pitching moments were stable, aiding pitch stability. Pressure distribution was reasonable, with good flow attachment and a rearward‐moving transition point, showing no premature stall. Consequently, the 10%–90% span range was selected for the coupled bionic design.

**Figure 8:**
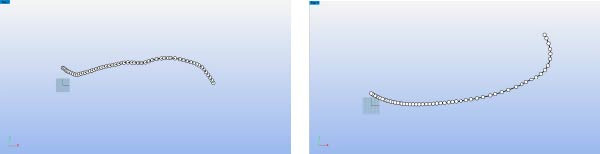
Leading edge curve and trailing edge curve.

**Figure 9:**
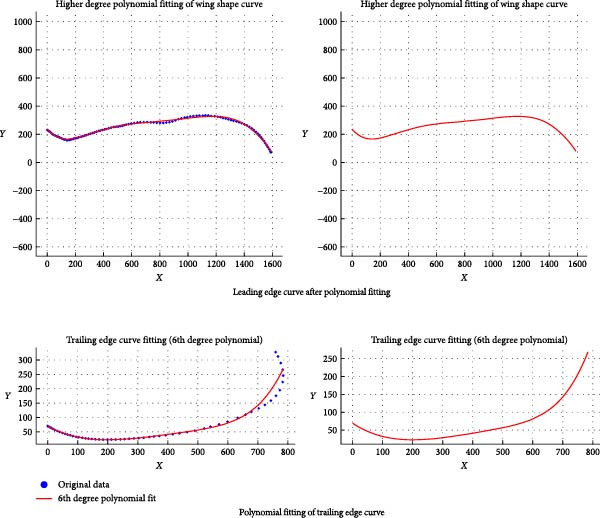
Polynomial fitting of leading and trailing edge curves.

Using the “Duplicate Edge” command in Rhino software, the leading and trailing edge curves of the Eurasian eagle‐owl wing were extract. Then, the “Add Points” command was applied to plot points on the leading and trailing edge curves, as shown in Figure 8. Their coordinates were then exported (TXT format). Polynomial fitting yielded the regression equations for the leading and trailing edges (Equations (20) and (21)). The fitted curves are shown in Figure 9.

The fitting relationship for the leading edge curve is presented in Equation (20):

(20) Y=−3.363.321.420.05521.093×10−10x4+×10−7x3−×10−4x2+x+.

The fitting relationship for the trailing edge curve is shown in Equation (21):

(21) Y=−1.3341.0523.0150.005350.1286×10−10x4+×10−7x3−×10−5x2+x+.

### 4.2. Construction of Coupled Extension Model Based on the Analysis of Eurasian Eagle‐Owl Wing Shape and Folding Wings of Flying Cars

Building on the coupled analysis methods for biomimetic design of flying car wings described in Section 3.2, an extension analysis of the coupled elements between the Eurasian eagle‐owl wing shape and the folding wings of flying cars was conducted. This included the integration of aerodynamic equations to perform extension analysis of coupling relationships. Further, a coupled extension model of the Eurasian eagle‐owl wing shape and the folding wings of flying cars was constructed. Based on this model, the coupled biomimetic design from the Eurasian eagle‐owl wing shape to the folding wings of flying cars was completed.

#### 4.2.1. Bio‐Coupled Element Extension Analysis

**Figure 10:**
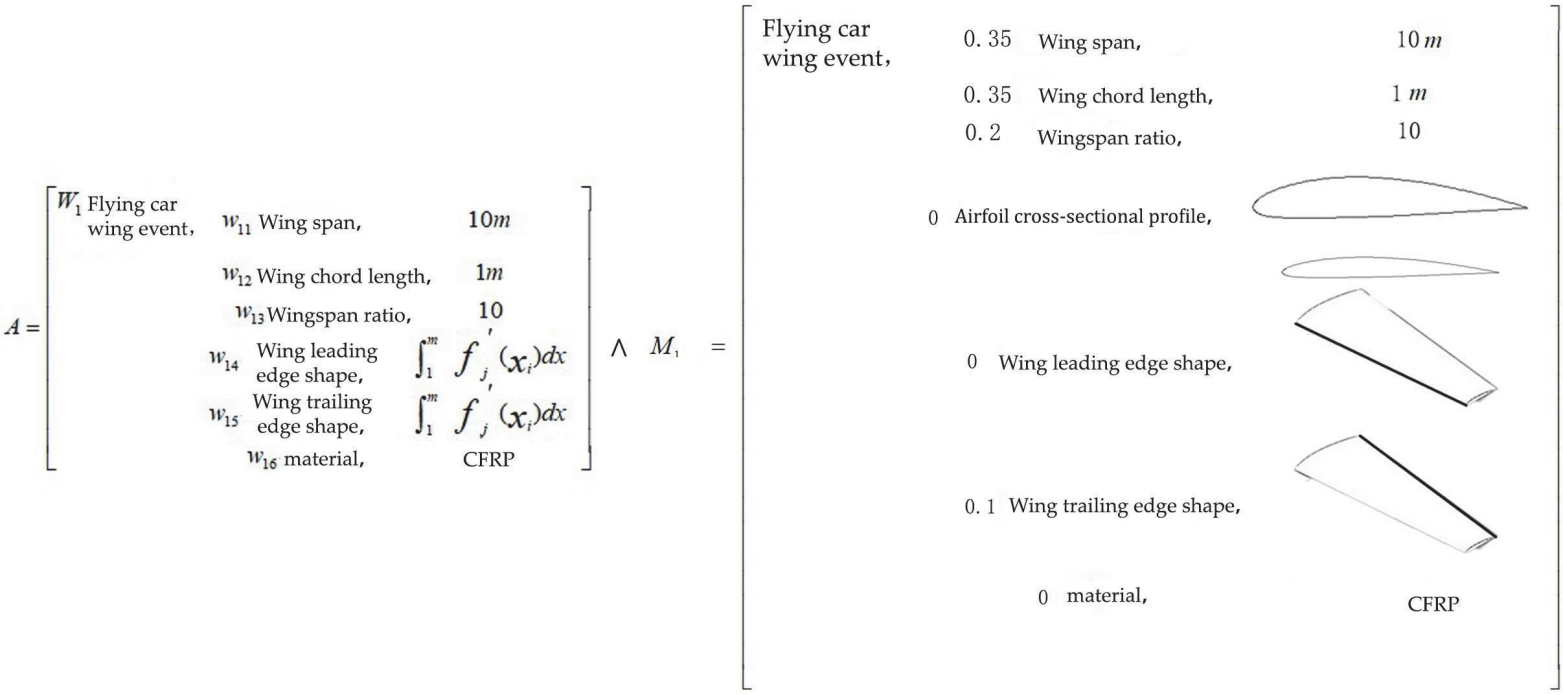
The morphological element matrix of the owl wing airfoil.

The extensible coupling process between the matter‐element and affair‐elements in the bionic design scheme for the folding wing of the flying car is represented as *A*∧*M*_1_∧*M*_2_. To determine the weight distribution of morphological features in the coupling bionic design, the G1 method was adopted to prioritize the evaluation of indicators within object‐elements *M*_1_ and *M*_2_. Ten experts with relevant professional backgrounds were invited to conduct a comprehensive evaluation using aerodynamic performance and functional compatibility of the folding wing as the core assessment criteria. Each morphological feature indicator was ranked and assigned a corresponding weight value. Based on the weight calculation formula, the final weight of each hierarchical indicator was calculated accordingly. According to the relationship *A*∧*M*_1_, the matter‐element of the coupling bionic design scheme for the new concept flying car was constructed. The corresponding extensible matrix is shown in Figure 10.

**Figure 11:**
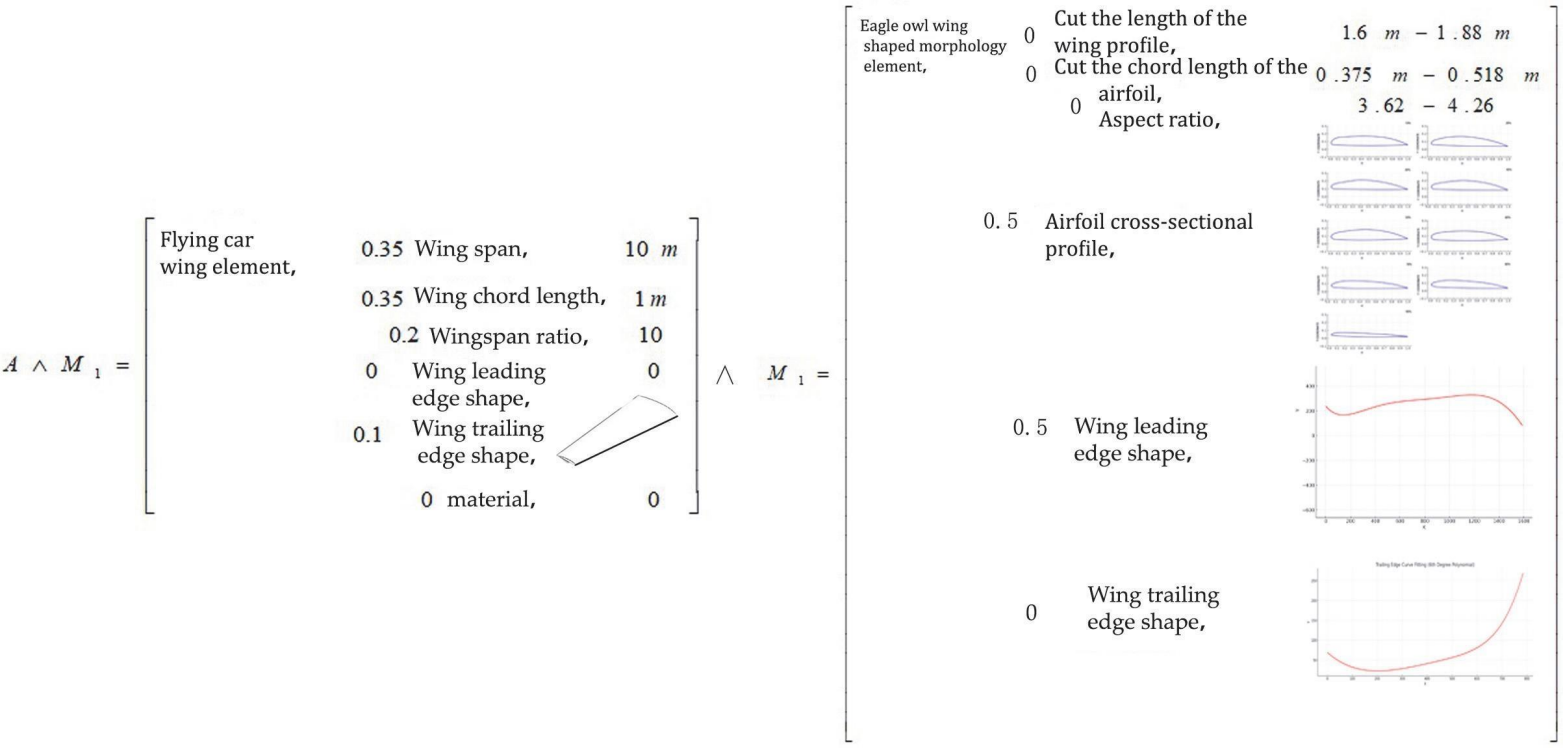
The functional element extensible matrix of the flying car wing.

Finally, the coupling with *M*_2_ was performed. The morphological coupling element data of the folding wing of the flying car, specifically wingspan, chord length, aspect ratio, and trailing edge shape, were coupled with the section profile and leading edge shape data of the barn owl wing. This formed the basis for the extension analysis of the characteristic vectors and eigenvalues of the folding wing matter‐element (extension matrix shown in Figure 11).

#### 4.2.2. Construction of the Coupled Extension Matrix Model

**Figure 12:**
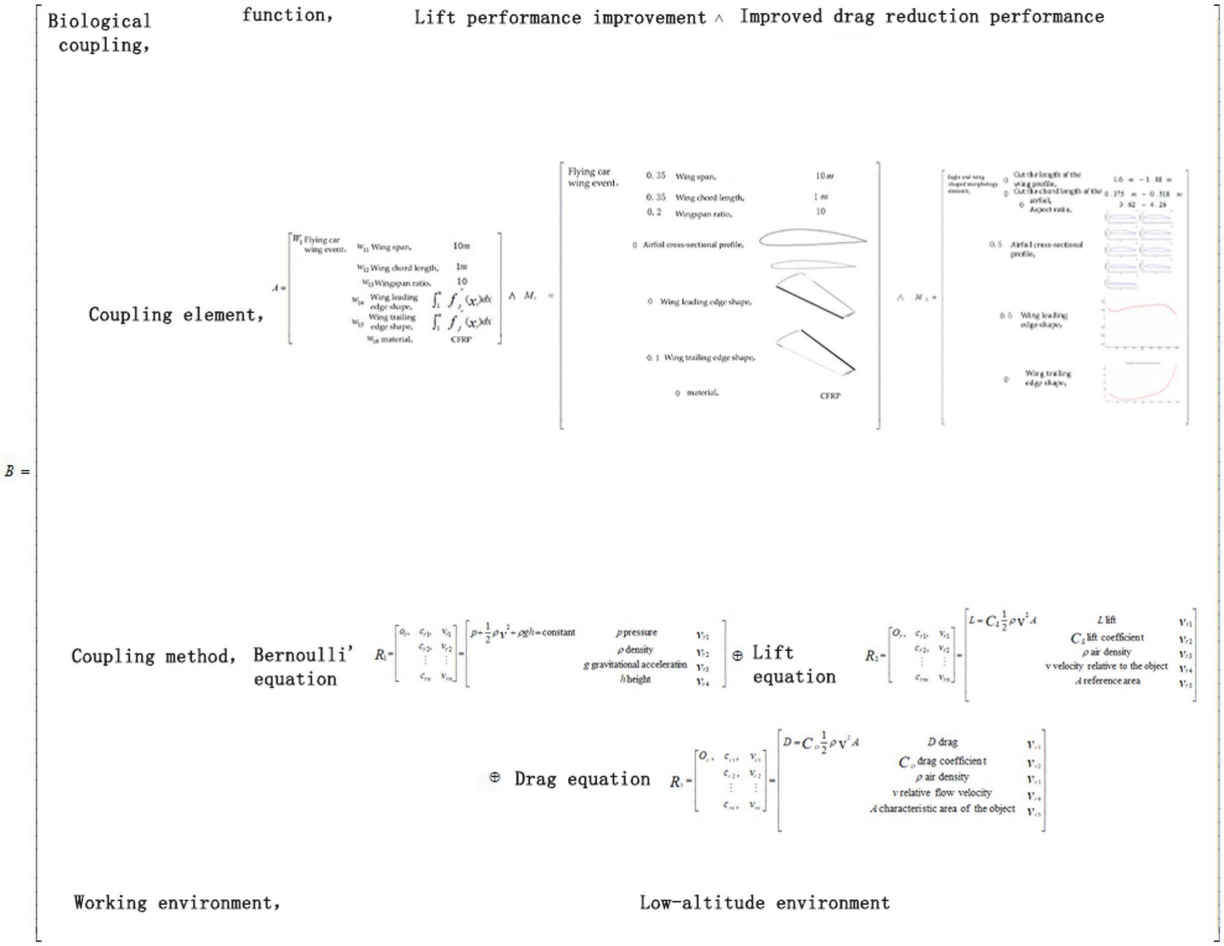
The coupled extensible data matrix of the owl wing airfoil and the folding wing of a flying car.

Based on the extension data of matter‐elements, matter‐elements in process (affair‐elements), and relation‐elements between the barn owl wing and the folding wing of the flying car, a coupling extension analysis was conducted between coupling element *A*∧*M*_1_∧*M*_2_ and the coupling modes. A coupling extension data matrix between the barn owl wing and the folding wing of the flying car was established, as shown in Figure 12. This matrix structure reflects the core design objective: enhancing the lift and drag reduction performance of the folding wing through biological coupling with the barn owl wing. By coupling the chord length, wingspan, aspect ratio, trailing edge shape, and material of the folding wing with the sectional contour and leading edge shape of the barn owl wing, a novel three‐dimensional design model of the folding wing was constructed. Under low‐altitude flight conditions, aerodynamic performance was comprehensively evaluated and optimized using the lift equation, the drag equation, and Bernoulli’s equation. Thus, a bionic coupling design and verification study of the flying car’s folding wing inspired by the barn owl wing was completed.

### 4.3. Coupled Bionic Design of Folding Wings for Flying Cars

**Figure 13:**
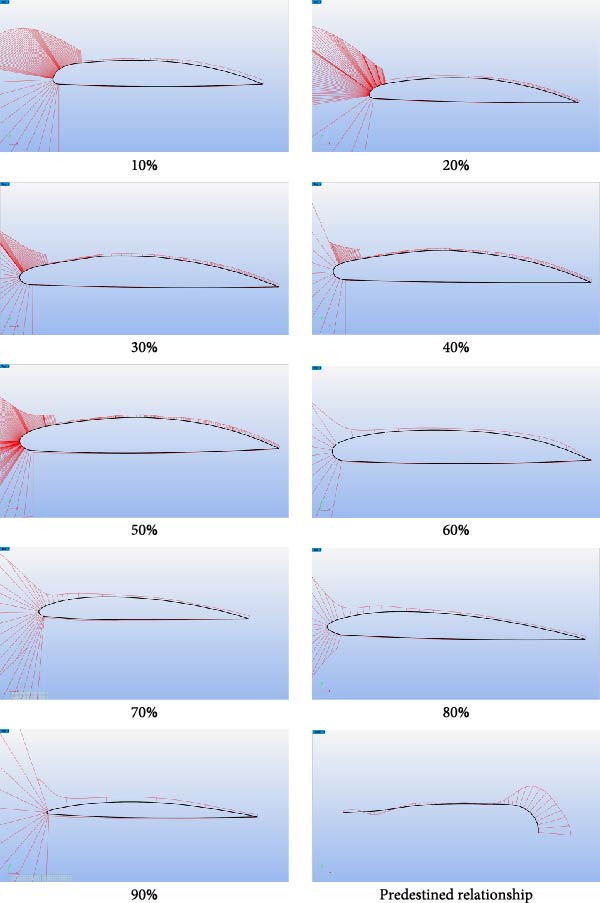
10%~90% airfoil curve, leading edge characteristic curve curvature analysis plot.

**Figure 14:**
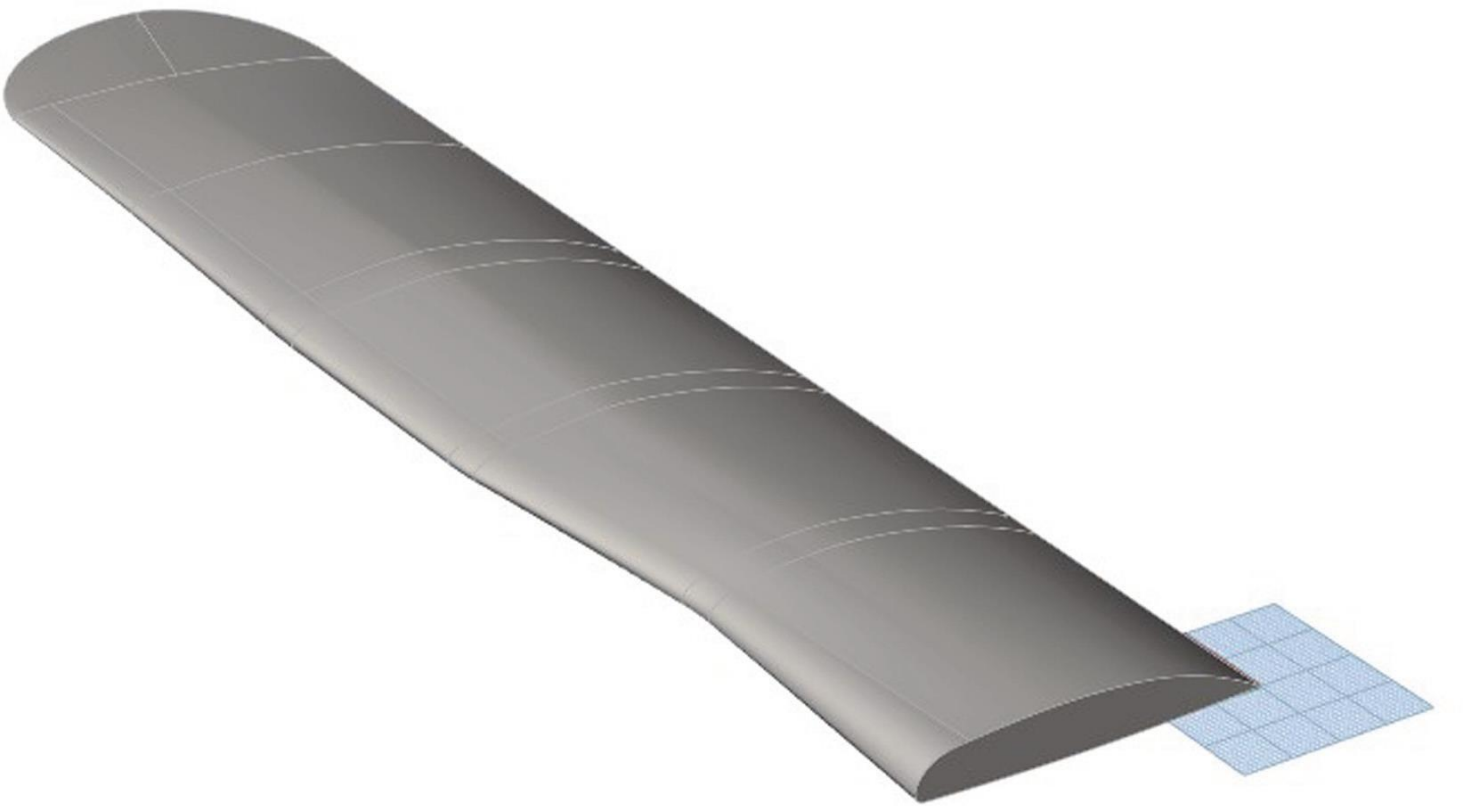
Three‐dimensional model of the flying car wing.

Based on the analysis of the bio‐coupled extension model, the coupled bionic design of folding wings for flying cars was conducted. The polynomial‐fitted cross‐sectional profiles and leading‐edge feature curves of the selected 10%–90% wing sections were saved in a Rhino‐compatible format and imported into the software. Within Rhino, non‐uniform rational B‐spline (NURBS) curves are created from this data. The curvature of these profiles and leading‐edge curves was analyzed using the “Curvature Graph Analysis” command, with calculated values displayed via a color map on the interface. Using this color map as a visual guide, the control points of the curves are adjusted iteratively until a smooth curvature distribution is achieved. Figure 13 shows the curvature analysis graph after adjusting the control points of the 10%–90% wing sections’ cross‐sectional profiles and leading edge feature curves. The curvature‐adjusted 10%–90% wing sections’ cross‐sectional profiles were scaled proportionally to a chord length of 0.9 m, and the leading edge feature curves were scaled to a half wingspan of 4 m. The trailing edge curves were designed to be straight, following the trailing edge of the Sharp Flight flying car’s folding wings under development. The 3D model of the wing was constructed using commands like “Unroll” and “Surface” in Rhino. Figure 14 shows the three‐dimensional model of the flying car wing.

### 4.4. Fluid Simulation Validation of Folding Wing Design for Flying Cars Based on the Eurasian Eagle‐Owl Wing Shape

#### 4.4.1. Determining the Computational Domain

**Figure 15:**
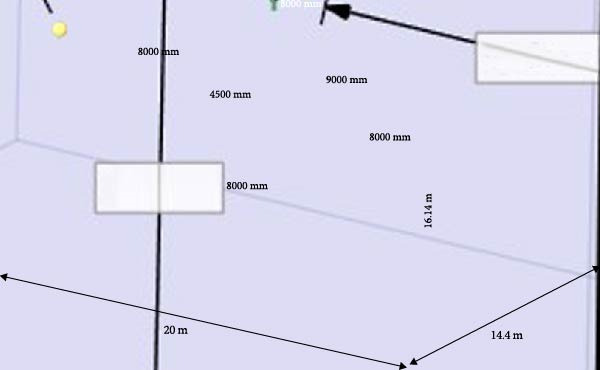
Computational domain of the flying car folding wing.

The wing model was imported into ANSYS Workbench via the “Geometry” module in SpaceClaim. The computational domain, a rectangular prism, was dimensioned to prevent flow disturbance, following the guidelines outlined by Jiri [19]. Letting c represent the maximum wing chord length and b the half‐span, the distances were set as follows: 5c from the wing leading edge to the velocity inlet, 10c from the trailing edge to the pressure outlet, and 2b from the wing to the top, bottom, and side boundaries. With *c* = 0.9 m and *b* = 4 m, the dimensions of the computational domain are: 20 m x 16.14 m x 14.1 m, as shown in Figure 15. The velocity inlet and the pressure outlet are the main boundary conditions. The velocity inlet requires a specified flow velocity to simulate real‐world operating conditions, while the pressure outlet is defined with a static pressure of 0 Pa (gauge) to realize smooth fluid exit. All other walls were set as no‐slip boundaries.

#### 4.4.2. Mesh Generation

**Figure 16:**
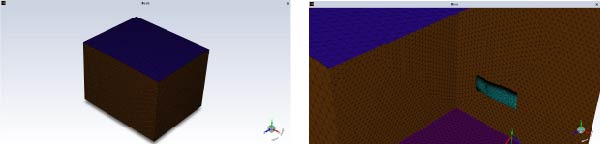
Wing mesh division.

The domain file was saved in. scdoc format and imported into Fluent Meshing software for mesh generation. An unstructured mesh was adopted to handle the complex geometry, with minimum and maximum sizes set to 0.1 mm and 500 mm, respectively. Mesh refinement was applied specifically at the leading and trailing edges to accurately capture flow features and boundary layer effects. Mesh quality was enhanced using refinement tools, ensuring a *y*+ value below 1 [24]. A mesh independence study was conducted to validate the results. The final mesh comprised 27,039,328 faces, as shown in Figure 16.

#### 4.4.3. Boundary Conditions and Turbulence Models

Flying cars typically operate in low‐altitude environments at relatively low speeds; therefore, the velocity inlet conditions for the inlet boundary in this study are set as follows: inflow velocities are set at 30, 40, 50, and 60 m/s; angles of attack are set at 0°, 2°, 4°, 6°, and 8° [25]. The air environment around the flying car wings uses an ideal gas model, which reduces the complexity of the simulation, with dynamic viscosity set at 1.7894e–5 kg/(m·s). The outlet boundary condition is set as a pressure outlet, with the pressure outlet set to 0 Pa (relative pressure). Setting the pressure outlet at 0 Pa helps simplify the numerical model and enhance computational stability and reduce numerical errors at the boundary, thus ensuring the accuracy of the simulation results. The surface of the flying car model and the boundaries of the flow field were set as no‐slip boundary conditions. In this study, to accurately simulate the complex flow field of the folding wings of a flying car under different flight conditions, the SST *k*–*ω* turbulence model was selected [26]. This model combines the accuracy of the k‐ε model in the near‐wall region with the robustness of the *k*–*ω* model in the far field, making it suitable for handling flow separation and reattachment phenomena around flying car wings.

#### 4.4.4. Numerical Calculation Results and Post‐Processing

A dynamic monitoring strategy was adopted to track the residuals of key variables in real‐time throughout the simulation process. These variables include mass (continuity), *X*‐velocity, *Y*‐velocity, *Z*‐velocity, turbulent kinetic energy (*k*), turbulence dissipation rate (*ε*), lift, and drag. The convergence criterion for residuals was set at 1 × 10^−4^, and calculations were run for 500 iterations. Fluent software output lift and drag data for the folding wing under various conditions (Table 4), along with pressure contours, velocity distributions, and streamline plots.

**Table 4:** Lift and drag related data of the flying car folding wing at different angles of attack and flow velocities.

Angle of attack (°)	Inflow velocity(m/s)	Lift coefficient	Drag coefficient	Lift–drag ratio
0°	30	0.39025398	0.02373463	16.44
	40	0.38918953	0.02341937	16.61
	50	0.39052589	0.02321065	16.82
	60	0.39181307	0.02305978	16.99

2°	30	0.44514805	0.02063297	21.57
	40	0.44630181	0.02026982	22.01
	50	0.44700927	0.02008698	22.25
	60	0.44738286	0.02003581	22.32

4°	30	0.53187775	0.01388189	38.31
	40	0.53239513	0.01358873	39.17
	50	0.5331235	0.01341342	39.74
	60	0.53361837	0.01337886	39.88

6°	30	0.56041551	0.0111276	50.36
	40	0.56111315	0.01082082	51.85
	50	0.56190362	0.01067033	52.66
	60	0.56727066	0.01086343	52.21

8°	30	0.61881416	0.01048837	59
	40	0.61925361	0.01032089	60
	50	0.61957196	0.01024085	60.51
	60	0.62005965	0.01016491	61.13

#### 4.4.5. Data Analysis

**Figure 17:**
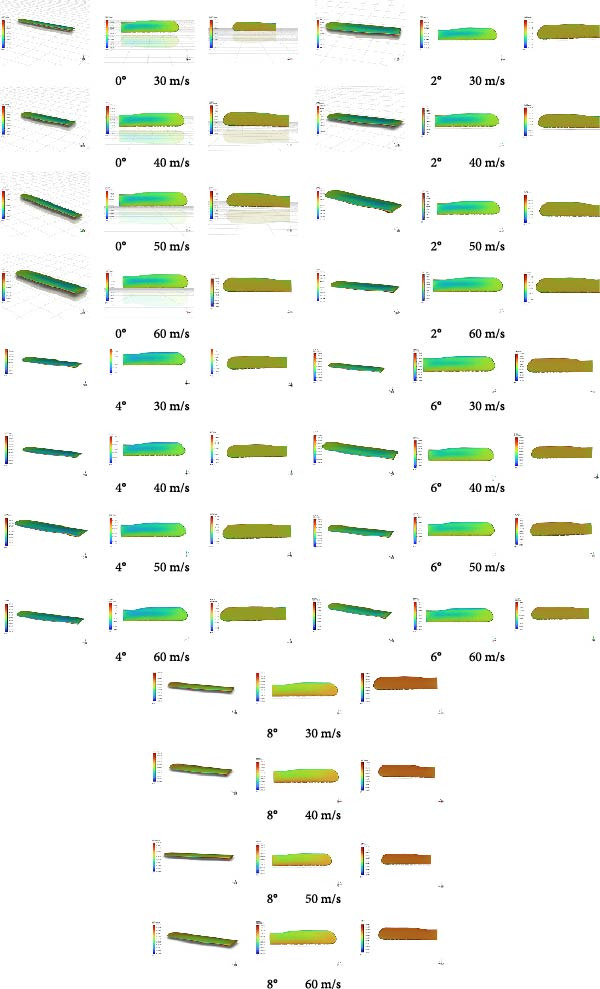
Static pressure distribution plot.

**Figure 18:**
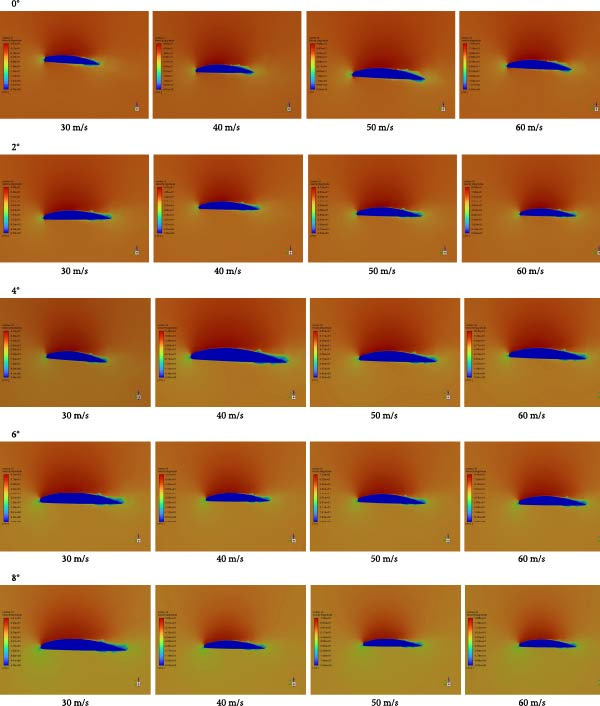
Speed contour plot of the flying car wing cross‐section.

**Figure 19:**
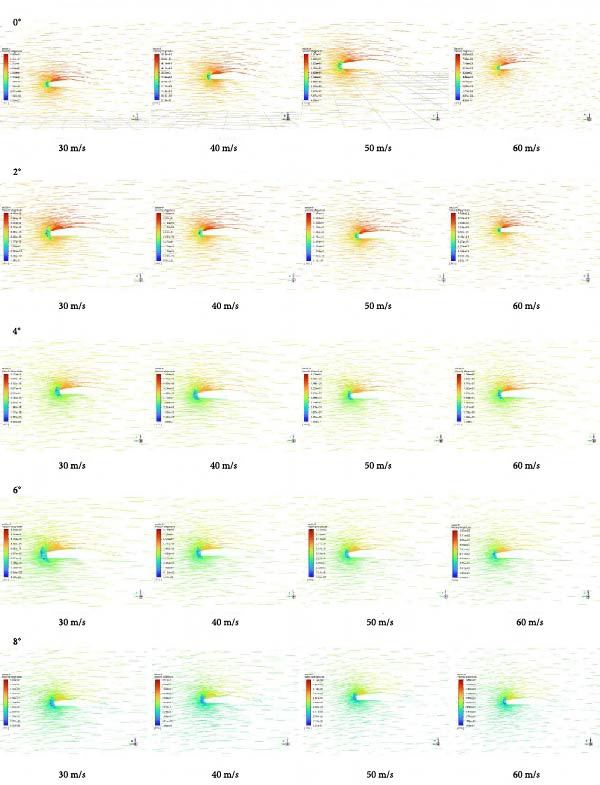
Speed vector plot of the flying car wing cross‐section.

Based on Table 5 (range values of lift‐to‐drag ratio for aircraft) [18], the LC, CD, and lift‐to‐drag ratio data of the folding wings of flying cars under various angles of attack and different flow velocities were analyzed. Using key flow field data obtained from Fluent (Table 4), the following findings were drawn: (1) Angle of attack significantly influenced the CL and CD, with an average change of + 11.9% in CL and −16.6% in CD across the range; (2) At a constant angle of attack, the LC exhibited a gradual increase across flow speeds of 30, 40, 50, and 60 m/s; the average increase rate was 0.06% at 0°, 0.15% at 2°, 0.06% at 4°, 0.4% at 6°, and 0.06% at 8°. Under the same angle of attack, the CD gradually decreased at these flow speeds; the average decrease rate was −0.9% at 0°, −0.9% at 2°, −1.2% at 4°, −0.9% at 6°, and −0.9% at 8°. (3) The LC indicated a clear positive correlation with the angle of attack and inflow speed, while the CD showed a clear negative correlation with these factors. (4) The lift‐to‐drag ratio significantly increased with increasing angle of attack and inflow speed, with values ranging from 16.44 to 61.13 from 0° to 8°, indicating that the wing’s coupled biomimetic design meets the lift‐to‐drag ratio standards outlined in Table 5. (5) In the case that flying cars were low‐altitude aircraft with relatively slow flying speeds, the wings required higher lift and smaller angles of attack. Comparing these standards to those of gliders is reasonable. Research data indicate that the wing’s coupled biomimetic design meets the requirements for the LC and CD of flying car wings. (6) The data related to lift and drag under different angles of attack and different flow speeds show a clear positive correlation with the pressure distribution data, velocity distribution data, and velocity vector map data, as shown in Figures 17–19.

**Table 5:** Range of the aircraft’s lift‐to‐drag ratio.

Type	Commercial aircraft	Glider	Fighter	Small private aircraft	UAV	Hypersonics
Lift‐to‐drag ratio range	15–20	40–60	5–10	10–15	8–12	3–5

## 5. Discussion

### 5.1. Discussion of Proposition Assumptions

This study proposes a hypothesis concerning the coupled bionic design and validation of flying car folding wings based on the Eurasian eagle‐owl wing shape. This proposition originates from efforts to improve upon the existing Ruixiang flying car folding wing design. Its theoretical foundation rests on coupled bionics theory and CFD. The basic data for the coupled biomimetic elements of the flying car’s folding wings are sourced from the wing type data of the sharp flight flying car folding wing scheme, while the coupled biomimetic element data are derived from the collected Eurasian eagle‐owl wing shape data.

### 5.2. Discussion of Method Design and Argumentation Process

The methodology follows a structured sequence. First, data collection involved selecting the Eurasian eagle‐owl wing as the biomimetic prototype through analysis of the Cornell Lab Bird Academy database. Subsequently, data for the Eurasian eagle‐owl wing shape was gathered through reverse engineering techniques, using image measurement methods combined with Meshroom and CATIA software, and multi‐angle two‐dimensional image data from the iNaturalist website to assemble a three‐dimensional point cloud data model of the wing shape. Finally, data preprocessing included equidistant slicing of the point cloud model, polynomial fitting of airfoil sections, and aerodynamic performance analysis to select optimal profiles.

Secondly, bio‐coupled extension analysis clarified the relationships between coupling elements (e.g., the flying car wing’s chord, span, aspect ratio, and trailing edge geometry coupled with the eagle‐owl’s airfoil section and leading‐edge shape), yielding the construction of a bio‐coupled extension matrix model. This model guided the 3D design of the folding wing.

Thirdly, CFD software was utilized to validate the fluid dynamics of the coupled biomimetic design of the flying car’s folding wings. The process began with defining the computational domain, followed by mesh generation, boundary condition setting, turbulence model selection, numerical simulation, and post‐processing and data analysis to obtain the fluid simulation validation results for the three‐dimensional model of the flying car’s folding wings design.

This method chain, including data collection, preprocessing, coupled extension analysis, and CFD validation, is scientifically sound, complete, and rigorous, ensuring the validity and credibility of the research outcomes.

### 5.3. Data Comparison

By comparing the fluid simulation data of the sharp flight flying car’s folding wings with those based on the Eurasian eagle‐owl wing shape, as shown in Table 6, the data indicates that at angles of attack of 0°, 2°, 4°, 6°, and 8°, and at incoming velocities of 30, 40, 50, and 60 m/s, the LC of the flying car folding wings based on the Eurasian eagle‐owl wing shape is improved, and the CD is significantly reduced compared to the sharp flight flying car’s folding wings. Empirical research demonstrates that the effectiveness of the coupled bionic design in enhancing wing performance, particularly in improving lift, reducing energy consumption, and enhancing flight efficiency.

**Table 6:** Comparison of lift coefficient and drag coefficient.

Eurasian eagle‐owl	Angle of attack	Inflow velocity	Lift coefficient	Drag coefficient	Ruixiang	Angle of attack	Inflow velocity	Lift coefficient	Drag coefficient
	0°	30	0.3902	0.0237		0°	30	0.3579	0.0283
		40	0.3891	0.0234			40	0.3584	0.0275
		50	0.3905	0.0232			50	0.3587	0.027
		60	0.3918	0.0230			60	0.359	0.0265
	2°	30	0.4451	0.0206		2°	30	0.3862	0.0277
		40	0.4463	0.0202			40	0.3868	0.0269
		50	0.4470	0.0200			50	0.3871	0.0264
		60	0.4473	0.0200			60	0.3868	0.0259
	4°	30	0.5318	0.0138		4°	30	0.4294	0.0266
		40	0.5323	0.0135			40	0.43	0.0258
		50	0.5331	0.0134			50	0.4307	0.0252
		60	0.5336	0.0133			60	0.4311	0.0248
	6°	30	0.5604	0.0111		6°	30	0.4437	0.0261
		40	0.5611	0.0108			40	0.4445	0.0253
		50	0.5619	0.0106			50	0.4452	0.0248
		60	0.5672	0.0108			60	0.4456	0.0243
	8°	30	0.6188	0.0104		8°	30	0.4721	0.0251
		40	0.6192	0.0103			40	0.4783	0.0247
		50	0.6195	0.0102			50	0.4788	0.0241
		60	0.6200	0.0101			60	0.4793	0.0237

### 5.4. Discussion of Pressure Contours, Velocity Distribution, and Velocity Vector Data

Based on the pressure contour as shown in Figure 16, combined with the evaluation vectors in Table 7, a comprehensive data analysis was conducted. The analysis reveals that at low angles of attack (0° and 2°) and speeds, pressure distribution is uniform with a slight increase at the wingtips. As speed increases, the pressure gradient from the wing root to the mid‐section significantly increases, indicating better airflow attachment and improved lift. At higher angles of attack (4°–8°), the pressure distribution remains relatively uniform at low speeds but shows increased gradients at higher speeds, particularly near the wingtips and trailing edge. Higher pressure at the root and leading edge promotes lift, but reduced pressure at the tips may decrease aerodynamic efficiency.

**Table 7:** Criteria for pressure contour plot, velocity distribution plot, and velocity vector plot.

Type	Evaluate vector
Pressure distribution plot	Evaluate based on the rationality of the pressure distribution, symmetry, the location of high and low‐pressure regions, smoothness of the pressure gradient, and boundary layer characteristics

Velocity distribution plot	Evaluate based on the symmetry of the velocity field, smoothness of the velocity gradient, flow separation, boundary layer distribution, and the rationality of high and low‐speed regions

Velocity vector plot	Evaluate based on the rationality of the velocity vector direction, smoothness of the velocity gradient, visualization of flow separation and vortices, boundary layer velocity distribution, flow field symmetry, rational distribution of high and low‐speed regions, and consistency of inlet and outlet velocities

Based on the velocity distribution map as shown in Figure 18, combined with the evaluation vectors in Table 7, a comprehensive data analysis was conducted. The analysis shows that at lower speeds (30 and 40 m/s) across all angles of attack, airflow adheres well to the wing surface with no significant separation, indicating high efficiency. At higher speeds (50 and 60 m/s) and higher angles of attack (4°–8°), velocity decreases at the wingtips, suggesting a risk of flow separation.

Based on the velocity vector map as shown in Figure 19, combined with the evaluation vectors in Table 7, a comprehensive data analysis was conducted. The analysis shows that at lower speeds (30 and 40 m/s), the airflow exhibits good adherence and smooth flow over the wing surface with no apparent flow separation or turbulence, indicating high aerodynamic efficiency and stable flow characteristics under these conditions. Notably at 0° angle of attack, the flow along the wing surface is very uniform, and the high‐velocity region formed at the leading edge further enhances airflow adherence. As speed increases to 50 and 60 m/s, at angles of attack of 4°, 6°, and 8°, the velocity near the wing tips decreases, and changes in flow direction begin to appear in the velocity vector map, indicating the onset of flow separation.

This comprehensive analysis confirms that the data from the flow field visualizations objectively reflect the aerodynamic design goals of the bionic wing model. While some reduction in efficiency and flow separation occurs at the wingtips under specific conditions, these effects are inherent consequences of the wing’s morphological design choices, remain within expected and controllable limits, and have a relatively low negative impact on overall flight stability and efficiency.

### 5.5. Research Innovation

1. Method innovation: This article constructs a tailored methodological framework for the biomimetic design of folding wings of flying cars specific to the research content of this paper. A particular innovation is the use of polynomial fitting equations to express the morphological feature curves of the Eurasian eagle‐owl wing shape during the coupled biomimetic design process. This allows for bio‐coupled extension analysis to determine characteristic vectors. By adjusting curve control points to ensure smoothness, adverse effects on aerodynamic efficiency in subsequent fluid simulations are minimized, achieving coupled biomimetic design of the flying car’s folding wings.
2. Innovation in the practical design of folding wings for flying cars: This research implements a practical bionic design for flying car folding wings grounded in coupled bionics theory and CFD. By mimicking the Eurasian eagle‐owl wing and integrating aerodynamic equations for lift and drag, the study produces a folding wing design that meets optimized shape requirements for aerodynamic performance, stability, safety, and adaptability to application scenarios.

### 5.6. Discussion on the Integration of Barn Owl Wing Morphology and Flying Car Folding Wing Design

During the validation process, the unique structural characteristics of the barn owl wing, such as serrated leading edges, flexible feather fringes, and active folding mechanisms, were identified. However, these localized features were deliberately excluded from the current bionic folding wing design for the following reasons.

First, from the perspective of aerodynamic contribution, the serrated leading edge is not a prominent morphological feature in the overall barn owl wing profile and has relatively limited influence on large‐scale wing surface lift or lift‐to‐drag ratio. Previous studies have shown that the serrated structure of the barn owl’s leading edge mainly functions under low Reynolds numbers and low‐speed flight conditions, contributing to effects such as boundary layer disturbance suppression and micro‐vortex control. These features serve primarily to achieve silent flight and improve airflow smoothness, with core functions focused on noise reduction and enhancing near‐field flow attachment, rather than significantly increasing lift or reducing drag over a broad area [27].

Second, regarding engineering feasibility, the folding mechanism of the barn owl wing is a biologically self‐organized process involving coordinated movement of flexible feather shafts, tendons, and multi‐joint articulations. This mechanism differs fundamentally from the rigid folding structures required in flying cars in terms of structural logic and actuation. Biological folding mechanisms exhibit high compliance, continuity, and nonlinear adaptability, whereas engineering folding systems prioritize controllability, limited degrees of freedom, structural rigidity, and dimensional constraints of the wing [28].

Therefore, given that this study focuses on aerodynamic optimization of the wing profile during the preliminary design stage for flying cars, the scope was intentionally limited to extracting the global contour of the barn owl wing and analyzing its macroscopic aerodynamic performance. The decision to exclude microscopic structures and flexible folding mechanisms was based on a comprehensive assessment of current data availability, research feasibility, and engineering modeling requirements.

In the process of bionic coupling analysis, the flexible properties of feathers indeed provide birds with excellent aerodynamic buffering and flow regulation capabilities during low‐speed flight, posture adjustment, and adaptation to airflow disturbances. Barn owl feathers, in particular, exhibit notable micro‐scale compliance and a multi‐layered flexible architecture, arising from hollow shafts, branched vanes, and hierarchical support structures.

However, from a biomimetic engineering perspective, translating the mechanical properties of flexible feathers into material parameters for flying car wing design faces several challenges. First, feather‐like flexible structures are suitable for small‐scale, lightweight aerial vehicles. In contrast, flying cars, as heavy‐duty transportation platforms, demand substantially higher material stiffness, safety margins, and fatigue strength, which far exceeds the performance limits of natural feather structures. Direct mimicry could compromise structural integrity and introduce safety risks.

Second, regarding material‐level coupling, flying cars, being high‐speed, passenger‐carrying flight platforms, demand wings with excellent engineering mechanical properties. Materials such as carbon fiber composites and aluminum alloys, known for their high specific strength, are typically used to ensure structural safety and load stability during flight. Although the flexible structure of barn owl feathers offers advantages in aerodynamic compliance and noise reduction, its mechanical performance falls short of the fundamental structural rigidity required by flying car applications. Moreover, under high‐load conditions, flexible materials may undergo uncontrolled deformation and delayed response, posing significant safety concerns.

Therefore, the decision to exclude the flexible material characteristics of feathers from the coupling bionic design path in this study is a rational constraint grounded in engineering adaptability and flight safety boundaries for flying cars [29].

### 5.7. Research Limitations and Future Research Directions

Equipment limitations constrained this study to numerical simulations for the fluid dynamics validation of the folding wing, and thus, the reliability of the results is subject to the accuracy and limitations of the Fluent software. In the reverse engineering process, the protected status of the barn owl (a Class II protected species in China) limited the use of physical specimen. As a result, the use of image‐based reverse engineering to reconstruct the wing morphology of the barn owl may have introduced a degree of inaccuracy in the results. This study focused solely on the geometric morphological features of the Eurasian eagle‐owl’s wing for the coupled bionic design of a flying car’s folding wing. It did not incorporate the owl’s structural or material characteristics into the bionic modeling and implementation, nor did it explore the integration of specific biological features, such as serrated leading edges or flexible feather structures, into folding mechanisms or material design.

Future work will involve the design of scaled physical models and wind tunnel testing to further validate the aerodynamic performance of the folding wing and advance practical applications. Subsequent studies should investigate the influence of the barn owl’s structural features and material properties on flying car wing performance, with particular emphasis on integrating biological traits like serrated leading edges and flexible feather‐inspired designs into structural and material bionics. The aim is to expand from morphology‐based biomimicry to a multilevel coupling approach that integrates structure and material, thereby deepening the scope of biomimetic research.

## 6. Conclusion

The research results demonstrate that the coupled biomimetic design of flying car folding wings based on the Eurasian Eagle‐Owl wing shape significantly improves the lift‐to‐drag ratio as the angle of attack and inflow velocity increase. The ratio ranges from 16.44 to 61.13 across angles of attack from 0° to 8°, meeting the lift‐to‐drag ratio standards. Given that flying cars operate at low altitudes and relatively slow speeds, their wings require high lift at small angles of attack; thus, using glider performance as a benchmark is justified. The findings confirm that the bionic wing design fulfills the specific objectives of enhancing the LC and reducing the CD. The three‐dimensional model of the coupled biomimetic wings effectively improves lift performance, reduces drag, enhances stability, and overall enhances the aerodynamic performance of the flying car wings. The research results validate the scientific rigor of the research methods and argumentation, advancing the theoretical and practical application of coupled bionics in the design of flying cars. The findings advance the design of folding wings for flying cars and hold definitive significance for engineering practice. This research underscores the value of theoretical application advancement, design efficiency enhancement, and engineering practical application.

## Disclosure

All authors have read and agreed to the published version of the manuscript and agree to be accountable for all aspects of the work. All content has been thoroughly reviewed and approved by the authors.

## Conflicts of Interest

The authors declare no conflicts of interest.

## Author Contributions

**Zhengjun Li:** conceptualization, methodology, formal analysis, supervision, writing – original draft, visualization. **Yuchen Cao:** conceptualization, investigation, writing – original draft, project administration, validation, visualization. **Dehao Zhao:** validation, visualization, writing – review & editing. Zhengjun Li and Dehao Zhao contributed equally to this work.

## Funding

The work submitted by the author has received support from the School of Design and Art, Shenyang Aerospace University. No funding was received to assist with the preparation of this manuscript. No funding was received for conducting this study. No funds, grants, or other support was received.

## Data Availability

The data that support the findings of this study are available from the corresponding author upon reasonable request.
